# Bladder and Bowel Management in Dogs With Spinal Cord Injury

**DOI:** 10.3389/fvets.2020.583342

**Published:** 2020-11-11

**Authors:** Nicolas Granger, Natasha J. Olby, Yvette S. Nout-Lomas, The Canine Spinal Cord Injury Consortium (CANSORT-SCI), Sarah A. Moore

**Affiliations:** Author Affiliations: Department of Veterinary Clinical Sciences, The Ohio State University College of Veterinary Medicine, Columbus, OH, United States; Department of Clinical Sciences, College of Veterinary Medicine, North Carolina State University, Raleigh, NC, United States; Department of Small Animal Clinical Sciences, College of Veterinary Medicine and Biomedical Sciences, Texas A&M University, College Station, TX, United States; Department of Veterinary Clinical Sciences, Purdue University College of Veterinary Medicine, West Lafayette, IN, United States; College of veterinary Medicine, Texas A&M University, College Station, TX, United States; Department of Veterinary Clinical Sciences, College of Veterinary Medicine, The Ohio State University, Columbus, OH, United States; Department of Clinical Sciences, Colorado State University, Fort Collins, CO, United States; Department of Clinical Science and Services, Royal Veterinary College, Hertfordshire, United Kingdom; The Royal Veterinary College, University of London, Hertfordshire, United Kingdom; CVS Referrals, Bristol Veterinary Specialists at Highcroft, Bristol, United Kingdom; Faculty of Veterinary Medicine, Institute of Veterinary Pathology, Leipzig University, Leipzig, Germany; Division of Clinical Neurology, Department for Clinical Veterinary Medicine, Vetsuisse Faculty, University of Bern, Bern, Switzerland; Department of Small Animal Medicine and Surgery, University of Veterinary Medicine Hannover, Hannover, Germany/Europe; Department of Veterinary Medicine and Surgery, MU Veterinary Health Center, University of Missouri, Columbia, MO, United States; Department of Small Animal Medicine and Surgery, University of Veterinary Medicine Hannover, Hannover, Germany/Europe; ^1^The Royal Veterinary College, University of London, Hertfordshire, United Kingdom; ^2^CVS Referrals, Bristol Veterinary Specialists at Highcroft, Bristol, United Kingdom; ^3^Department of Clinical Sciences, North Carolina State University College of Veterinary Medicine, Raleigh, NC, United States; ^4^Department of Clinical Sciences, Colorado State University, Fort Collins, CO, United States

**Keywords:** bladder, urinary and fecal incontinence, spinal cord injury, autonomic, dysfunction, canine, dog, sacral implant

## Abstract

Spinal cord injury in companion dogs can lead to urinary and fecal incontinence or retention, depending on the severity, and localization of the lesion along the canine nervous system. The bladder and gastrointestinal dysfunction caused by lesions of the autonomic system can be difficult to recognize, interpret and are easily overlooked. Nevertheless, it is crucial to maintain a high degree of awareness of the impact of micturition and defecation disturbances on the animal's condition, welfare and on the owner. The management of these disabilities is all the more challenging that the autonomic nervous system physiology is a complex topic. In this review, we propose to briefly remind the reader the physiology of micturition and defecation in dogs. We then present the bladder and gastrointestinal clinical signs associated with sacral lesions (i.e., the L7-S3 spinal cord segments and nerves) and supra-sacral lesions (i.e., cranial to the L7 spinal cord segment), largely in the context of intervertebral disc herniation. We summarize what is known about the natural recovery of urinary and fecal continence in dogs after spinal cord injury. In particular we review the incidence of urinary tract infection after injury. We finally explore the past and recent literature describing management of urinary and fecal dysfunction in the acute and chronic phase of spinal cord injury. This comprises medical therapies but importantly a number of surgical options, some known for decades such as sacral nerve stimulation, that might spark some interest in the field of spinal cord injury in companion dogs.

## Introduction

Spinal cord injury can cause irreversible locomotor and autonomic dysfunction including urinary and fecal incontinence. The two functions of the bladder and bowel are *storage* and *voiding*. After severe spinal cord injury, both of these are impaired as a result of altered sensation and altered voluntary control amongst others. People with thoracic and thoracolumbar spinal cord injury value restoration of bladder and bowel control by far over regaining walking (1, 2), which signifies the great social and psychological consequences of urinary and fecal incontinence. In dogs, the impact of urinary and fecal incontinence on quality of life is not clear but lack of recovery of continence affects owners and increases the time and demands required to care for dogs (3). It seems that the focus of owners is regaining locomotion, and that urinary or fecal incontinence, whether persistent or temporary, are forgotten or “managed” dysfunctions that will either pass or will need to be accepted as permanent. Further, the problem for owners caring for a dog with urinary and fecal incontinence varies greatly depending on the localization of the inciting injury: (i) with low lumbar lesions, the incontinence is often permanent and consistent, therefore more problematic, due to loss of sphincter tone, which likely results in a greater demand for euthanasia; (ii) with “supra-sacral” lesions, there is retention urinary incontinence characterized by preserved or increased sphincter tone and involuntary bladder contractions (i.e., reflex incontinence) and fecal incontinence characterized by inability to control defecation once the defecation reflex has been triggered; therefore the owner's challenges are typically those of caring for a dog where manual emptying of the bladder is difficult, there is regular emission of small volumes of urine and there is sudden urge fecal incontinence (4). The treatment goals to address these difficulties are very different, sometimes requiring stimulating a function sometimes requiring blocking a function, and this adds to the complexity of managing bladder and bowel dysfunction in animals.

In this paper, we will review briefly the physiology of micturition and defecation, the characteristics of urinary and fecal incontinence after spinal cord injury as well as their recovery, and we will assess the extent to which these events occur in spinal cord-injured dogs. We will provide state of the art management recommendations for bladder and bowel dysfunction in the acute (from the day of injury to approximately a month) and chronic (the weeks to months after injury) phases of spinal cord injury in dogs and discuss these related to lesion level. We will also identify aspects of autonomic dysfunction that are currently unclear in spinal cord injured dogs and provide opportunities for further studies. This could be critical to better understanding and manipulating these systems and therefore providing avenues for development of therapeutic strategies for dogs and humans via translational mechanisms.

## Physiology of Micturition

Here we provide a brief overview of the physiology of micturition; for a detailed review on anatomy and physiology the reader is referred elsewhere (5–8).

Regulation of urination involves a complex neural control system in the brain, spinal cord, and peripheral autonomic ganglia that coordinates the activity of smooth and striated muscles of bladder and urethral outlet. Spinal storage mechanisms are regulated by circuitry in the rostral brain stem and initiate reflex voiding. Input from the forebrain triggers voluntary voiding by modulating the brainstem circuitry.

The switch between storage and voiding is mediated by a long-loop spino-bulbo-spinal voiding reflex which has its rostral terminus in the brainstem. During urine storage, as the bladder fills, bladder afferent signals increase in strength until they exceed a certain threshold in the brainstem. In the absence of any controlling influences, the pontine micturition center is activated, the urethral sphincter relaxes, the bladder contracts, and reflex voiding occurs (8). When the bladder is empty, urine storage resumes. Cortical modulation of this circuitry underlies the voluntary control of voiding. In addition, neurons in other subcortical areas (e.g., the *nucleus subceruleus, reticularis pontis oralis*, cerebellum, hypothalamus, medullary raphe nucleus) exert direct or indirect modulatory influences on the voiding reflex (8).

Micturition involves the parasympathetic system (pelvic motor and sensory neurons and nerves), sympathetic system (hypogastric motor and sensory neurons and nerves) and somatic pathways (from the brain to the sacral spinal cord segments and pudendal nerve). Sensory input on bladder filling is transmitted via the pelvic nerve from the detrusor (smooth) muscle to sacral spinal cord segments (9, 10) and via ascending spinal cord tracts to the pontine reticular formation. The pontine reticular formation is in turn responsible for the micturition reflex through activation of parasympathetic influence (pelvic nerve) and reduction of sympathetic influence (hypogastric) and inhibition of sphincter muscle contraction (pudendal) (8, 11). This constitutes the detrusor reflex. Sensory input also travels to the cerebrum which strongly influences detrusor muscle activation during micturition in normal animals (8, 12). Furthermore, the cerebellum has an inhibitory influence on detrusor muscle activation. Altogether these systems allow for full voluntary bladder emptying.

Urine storage is facilitated by the urethral sphincter. Stretch at that level is sensed by the spindle cells of the striated muscles of the urethral sphincter and transmitted via the pudendal nerve to the sacral spinal cord segments (L7-S3; motoneurons of the pudendal nerve are located within Onuf's nucleus in the ventral horn of the sacral spinal cord). Once the stretch threshold has been reached there is a direct (monosynaptic) motor response via the pudendal nerve activating the external urethral sphincter composed of striated muscle fibers leading to sphincter closure. This motor response also occurs when there is firing from the pelvic nerve afferents allowing continence in situations when there is sudden increase in bladder pressure (e.g., when jumping, barking etc.). The external component of the urethral sphincter is also under influence of the cortex, allowing voluntary contraction or inhibition, and the pelvic nerve, which during detrusor muscle contraction inhibits pudendal nerve firing (8).

Finally, storage of urine is facilitated by the hypogastric nerve, which originates from the L1 to L4 spinal cord segments in the dog. The alpha-adrenergic component synapses to the bladder trigone, neck and proximal urethra and causes contraction. The beta-adrenergic component synapses to the detrusor muscle and allows smooth muscle relaxation. The hypogastric nerve feeds back to the brain information on bladder overdistension and bladder pain signals during inflammatory processes such as urinary tract infection (8), thereby providing a protective mechanism which may be lacking after spinal cord injury. During voiding, pelvic nerve neurons inhibit hypogastric neurons.

## Physiology of Defecation

Here we provide a brief overview of the physiology of defecation; for a detailed review on physiology and pathophysiology after spinal cord injury the reader is referred elsewhere (13, 14) and De Lahunta and Glass also provide an overview in companion animals in their textbook (6).

The normal neurophysiological control of the gastrointestinal system is dependent on local enteric circuits, autonomic input through the parasympathetic and sympathetic nervous systems, and higher cortical processes that serve to control timing of elimination. The local regulation of many gastrointestinal reflex functions is governed by enteric neurocircuitry that is capable of independent secretory and motor (propulsive) reflexes as well as regulating the homeostatic requirements of the gastrointestinal tissues such as blood flow (13, 15). Parasympathetic innervation to the descending colon and rectum arises within the sacral spinal cord segments and travels by way of the pelvic nerve, promoting motility. Gastrointestinal sensory input is derived by way of the hypogastric nerve originating in the lumbar spinal cord segments (L1 to L4 in dogs) and hypogastric nerve stimulation elevates internal anal sphincter pressure and inhibits the descending colon and rectum. Sympathetic input is largely inhibitory of motor and secretory processes and provokes vasoconstriction. Transmission and perception of visceral nociceptive stimuli is generally considered to be relayed through the sympathetic splanchnic nerves and terminates within the spinal cord. Voluntary control and closure of the external anal sphincter muscle is through the pudendal nerve of which its motoneurons are located within Onuf's nucleus. This system is under cortico-thalamic and brainstem control through ascending (dorsal, dorsolateral, and ventrolateral white matter tracts) spinal tracts and descending reticulo-spinal tracts.

During the storage phase, there is autonomic unconscious closure of the internal (hypogastric nerve control) and external (pudendal nerve control) anal sphincters while the descending colon and rectum are inhibited (hypogastric nerve control). When the rectum is full, there is involuntary relaxation of the internal anal sphincter (recto-sphincteric reflex) and defecation can then occur when decided (16). This involves cortically driven relaxation of the external anal sphincter (pudendal nerve control), contraction of abdominal wall muscles and relaxation of pelvic wall muscles.

## Consequences of Spinal Cord Injury on Micturition

We make a distinction through the rest of the text between urinary incontinence defined as involuntary loss of urine and urinary retention defined as the inability to voluntary void.

### Supra-Sacral Lesions

Lesions cranial to the L7 spinal cord segment result in preservation of the sacral spinal cord segments but disruption of the ascending and descending pathways to and from the brain. This loss of supraspinal input results in impaired control over detrusor muscle activation and relaxation and loss of bladder fullness sensation and voluntary micturition. The pelvic and pudendal nerves remain functional; therefore, the external urethral sphincter remains closed. This condition is often referred to as “upper motor neuron” bladder, and clinically, the manifestation is urine retention with a large distended bladder, often felt as tensed or firm on palpation, that is difficult to express. Some neurologists have suggested presence of “small unsynchronized” bladder contractions (5). Therefore, there is incontinence either due to bladder overflow or intermittent emission of urine from involuntary bladder contractions. These animals are at risk for bladder rupture either through excess filling against a closed sphincter or as a complication during manual bladder expression, although this is rare.

### Sacral Lesions

Sacral lesions result in loss of bladder innervation from the pelvic and pudendal nerves which causes loss of detrusor muscle function and urethral sphincter tone. It results in excessive bladder distension that feels flaccid on manual palpation and there is typically constant leakage of urine due to poor sphincter function. This is often referred as “lower motor neuron” bladder. The incontinence can occur because of overflow or simply when the pressure in the bladder exceeds that of the weakened urethral sphincter but clinically, the bladder is often quite large. The animal is frequently soiled with urine and this causes major difficulties in nursing care of these patients in hospital and also at home.

In some instances of focal sacral lesions, sphincter tone can be preserved resulting in a combination of flaccid detrusor muscle with sphincter tone that is difficult to overcome and resulting bladder distension. While this is most commonly seen in cats with “tail pull” injuries that damage the pelvic and pudendal nerve roots, it can occur with dogs that have suffered a focal sacral lesion due to acute non-compressive nucleus pulposus extrusions or fibrocartilaginous emboli (17). The exact mechanisms causing this type of dysfunction remain unclear, although there is a suggestion that this could be mediated by the preserved hypogastric structures and internal urethral function.

### Is Spinal Shock Associated With Bladder Atony?

Following transection of the spinal cord in experimental dogs (between T8 and T12), a state of spinal shock occurs immediately and leads to complete urinary retention and areflexia of the detrusor for a period of 2–6 weeks (18, 19) before reflex detrusor activity returns. Spinal shock is also known to occur in some companion dogs after “natural” severe spinal cord injury (20, 21) and leads to loss of spinal reflexes, sensation and muscle tone below the lesion for a period of time, usually ~24–48 h. It is not clear in companion dogs whether spinal shock in cases with severe, acute spinal cord injury also leads to prolonged loss of sacral reflexes and bladder atony and there is limited data on that topic. Atalan et al. (22) described a gradual return of normal residual volume in 25 dogs recovering from spinal cord injury. Levine et al. (23) showed that bladder compliance, capacity, and residual volume were higher in 20 dogs with natural occurring acute spinal cord injury from the day of presentation to 3, 7, and 42 days after spinal cord injury than their control dogs but the presence or absence of spinal shock was not mentioned; however, the control group consisted of normal experimental dogs and not companion dogs and it is unknown whether these dogs have similar bladder function to house trained dogs. The finding of Levine at al. that bladder tone is increased in acute spinal cord injury (23) is opposite to the finding in experimental dogs where the bladder is atonic in the acute phase after spina cord transection (18, 19). However, this clinical trial recruited dogs with Hansen type 1 intervertebral disc herniation in which spinal shock is less common than in more severe cases, moreover, the majority had only moderately severe injuries, again making spinal shock unlikely. In another study on thoracolumbar acute non-compressive nucleus pulposus extrusion, a weak association was found between spinal shock and the odds of remaining faecally incontinent, although this likely reflects an association between spinal shock and severity of injury (24). Interestingly, bladder atony at the time of spinal shock can be improved with functional electrical stimulation of sacral nerve roots in experimental dogs (25, 26) when delivered early in the disease process. Levine et al. (23) also showed that early pharmacological intervention with sub-cutaneous injection of a matrix metalloproteinase inhibitor could improve bladder compliance. These findings form interesting observations on which we can build further studies. In particular, we need to define whether bladder atony occurs in the most severe cases of canine spinal cord injury (i.e., with complete loss of sensori-motor function and loss of deep pain). We then need to study the sustainability of the effect of early bladder interventions (in particular functional electrical stimulation) on continence, particularly in those cases that will not recover deep pain.

## Consequences of Spinal Cord Injury on Gastrointestinal Function

With upper motor neuron lesions (i.e., supra-sacral) the external anal sphincter remains closed and feces accumulate. The animal occasionally drops feces without awareness when the pressure in the colon and rectum increases and causes involuntary reflex evacuation. Fecal incontinence is largely a feature of thoraco-lumbar (T3-L3) lesions causing paraplegia since traumatic cervical injuries in dogs severe enough to cause fecal incontinence are extremely rare (4, 27). Companion dogs with only moderate gait deficits and concomitant fecal incontinence have been infrequently reported and shown to have discrete lesions in the dorsal aspect of the spinal cord. These cases are rarely diagnosed with intervertebral disc herniation (28, 29). The most prevalent gastrointestinal co-morbidity following spinal cord injury in humans is “neurogenic bowel”, which is frequently described as colonic dysfunction that presents as reduced colonic contractions and transit, constipation, disordered evacuation reflexes and potential overflow incontinence. In dogs, constipation is rarely a problem, but it is encountered in cats following spinal cord injury more commonly (30). Also, in humans and rat there is evidence for development of esophageal and gastric impairment of function after upper spinal cord injury (with increased incidence of heartburn and esophageal chest pain chronically) (31). In dogs, subclinical gastroduodenal ulceration has been reported in only one study with a prevalence as high as 76%, raising awareness of that possible complication in the acute phase of intervertebral disc extrusion (32). Finally, the development of autonomic dysreflexia, a condition in which a noxious visceral stimulation, often accompanying severe constipation or bladder distension, triggers a life-threatening increase in sympathetic discharge below the injury level and systemic hypertension, has so far not been shown to occur in dogs. In humans and rodents this syndrome is seen to occur secondary to severe cervical and high thoracic lesion and can be fatal but high thoracic lesions are rare in dogs, so it is difficult to know if autonomic dysreflexia exists or not in dogs.

Fecal incontinence is a more common problem in dogs that suffer acute contusive or vascular (i.e., “non-compressive”) lesions of the spinal cord, such as acute nucleus pulposus extrusion or fibro-cartilaginous embolism, respectively (24, 33) compared to compressive lesions. This might be because parenchymal lesions are more centrally located within the spinal cord or perhaps resulting from dilation of the spinal canal. This is seen in dogs with chronic compression of the spinal cord from sub-arachnoid diverticulum, such as in Pugs where fecal incontinence is common. The prognosis for dogs with acute contusive or vascular lesions is further discussed in the treatment section below.

In cases with spinal cord injury caused by intervertebral disc extrusion (i.e., a “compressive” lesion) in the T3-L3 spinal cord segments, ~40% of owners of dogs that have recovered from paraplegia with loss of deep pain report that their dog's fecal continence is not as good as it was prior to their injury (4, 27). They report two types of fecal incontinence. In the first type, called sudden urge incontinence here, the dog becomes acutely aware of the need to defecate, makes a dash for a suitable location and is unable to prevent the defecation reflex. The second type occurs when the dog drops stool without apparently being aware of it. Both of these forms of incontinence can happen to differing degrees in dogs that are also able to defecate voluntarily. For most owners, this is not a major complaint, but for a small subset the severity of the problem results in euthanasia of the dog whether the dog has recovered motor function or not (4).

With lower motor neuron lesions (i.e., sacral), the anal sphincter is weak or absent and this causes constant leakage of feces. This is a much more serious problem to manage the dog in hospital and at home and can lead to megacolon in the author's experience.

## Spontaneous Recovery of Urinary and Fecal Continence After Spinal Cord Injury

### Possible Mechanisms Underlying Recovery

Reorganization of the micturition reflex following spinal cord injury is dependent in part on the plasticity of bladder afferent pathways and the unmasking of reflexes triggered by unmyelinated, capsaicin-sensitive, C-fiber bladder afferent neurons. Plasticity of bladder afferent neurons is associated with morphologic, chemical, and electrical changes, which appears to be mediated in part by neurotrophic factors released at the level of spinal cord and the peripheral target organs (34). Upregulation of anti-inflammatory mediators and neuroprotective molecules is likely to play an important role in the plasticity of bladder afferent pathways as well as reorganization of synaptic connections in the spinal cord (35). In rats, poor voiding efficiency at 4 and 8 weeks after spinal cord injury was coincident with upregulation of pro-inflammatory cytokines (IL-1α, IL-1β, IL-2 IL-5, IL-6, IL-18, and TNF-α), chemokines (CX3CL-1, CCL2, CXCL-1, CXCL2, CXCL-10) and downregulation of anti-inflammatory cytokines IL-4 and IL-13, whereas spontaneous recovery of voiding function at 12 weeks was associated with maximum expression of anti-inflammatory cytokine IL-1018, neurotrophin BDNF and CXCL-5 as well as the neuroprotective leptin19 in bladder (16).

Similarly, the negative impact of inflammation in the recovery process might be greater than previously thought, opening some possible research avenues to look at controlling this inflammatory response. Multiorgan dysfunction following spinal cord injury has been recognized to start minutes to weeks after spinal cord injury with both visceral and somatic tissues affected, including the cardiovascular, pulmonary, renal, skeletomuscular and hepatic systems (36). Also, neuroendocrine changes along the hypothalamic-pituitary-adrenal axis elevate circulating macrophage migration inhibitory factor. It is likely that the systemic inflammatory response that is initiated at the spinal cord injury site, spills over to the circulation, contributing further to acute-phase hepatic pro-inflammatory release.

### Clinical Characteristics of the Recovery

With intervertebral disc herniation causing compression within the T3-L3 spinal cord segments (upper motor neuron lesions) and when dogs have present (intact) deep pain sensation in the pelvic limbs, recovery of urination usually occurs concomitantly with return of motor function (5, 22, 23) and the prognosis for recovery of urinary continence is good (17). When deep pain sensation is absent at the time of injury, the prognosis for recovery is less certain (4, 27) and ~50–60% of dogs eventually regain urination along with deep pain and walking. Of these dogs recovering, ~30% of owners report less than perfect urinary continence. In particular, they report that they cannot leave their pet without access to somewhere to urinate for as long as they could before the injury and find that the excitement of their return to the house will trigger involuntary urination (4). Further, 30% (27/93) to 59% (48/81) of dogs that do not recover pain perception after intervertebral disc herniation can develop “spinal” walking (i.e., an automatic gait lacking presumably brain control) but none of these spinal walking dogs fully recover either urinary or fecal continence (4, 27, 37). Urinary continence has not been evaluated in detail in these dogs, but it is clear that reflex voiding is present with varying degrees of success and efficiency of bladder emptying, starting in the weeks after injury. Animals with no deep pain can also develop ascending/descending myelomalacia in the days after spinal cord injury (38, 39); in a subset of cases, there is only descending myelomalacia (or the ascending lesions stop progressing and the front limb function is spared while there is descending myelomalacia) and this can lead to destruction of the sacral spinal cord segments and lower motor neuron incontinence and, in our experience, rectal prolapse, with a poor prognosis.

The prognosis for recovery of urinary continence in dogs with non-compressive T3-L3 spinal cord injury seems similar to those with a compressive injury, with 91–98% of dogs with hind limb dysfunction and present (intact) deep pain regaining urinary continence (24, 40, 41). However, interestingly, 15–23% of dogs remain faecally incontinent in the chronic phase of recovery (24, 40, 41), suggesting that the injury perhaps affects spinal cord tracts differently to acute “compressive” intervertebral disc herniation. Indeed, contusive and vascular lesions induce more centrally located damage (as evident on MRI) than is induced by compressive intervertebral disc extrusion, likely affecting the descending inhibitory control of the defecation reflex. The possibility of persistent fecal incontinence should be conveyed to owners of these cases.

With intervertebral disc herniation causing compression of the lumbo-sacral spinal cord segments (L4 to S3) (lower motor neuron lesions) or in dogs with non-compressive lesions of these segments, data on recovery are scarce. A recent study looked at the recovery rate within the first 3 weeks after injury (17). Dogs over 15 kg with compressive lesions and lower motor neuron bladder or dogs with non-compressive lesions and upper motor neuron bladder (e.g., dogs with lesions of the lumbar spinal cord segments rather than sacral) recovered urinary continence in ~60–70% of the cases. However, dogs with non-compressive lesions and lower motor neuron bladder (i.e., lesions likely extending from the lumbar spinal cord segments to the sacral segments) never recovered continence in that study, although this only represented 5 dogs. Finally, in 13 dogs with absent deep pain and a lesion within the L4-S3 spinal cord segments, only one dog recovered continence, signaling a poor prognosis for this category of dogs.

Secondary lesions of the lower urinary tract in the acute and then chronic phases of spinal cord injury are poorly described in companion dogs. There is a clear occurrence of urinary tract infections (bacterial cystitis, pyelonephritis) that is discussed alongside the management methods to drain the bladder in the following parts of this article. But the histopathological changes of the bladder (e.g., bladder fibrosis or loss of the neuro-muscular junction at the detrusor level) or kidneys (e.g., vesico-ureteral reflux and hydronephrosis) are not described in companion dogs although there are data in experimental dogs (42, 43).

## Management of Urinary Retention and Incontinence in the Acute Phase and Early Weeks After Spinal Cord Injury

Diligent management of retention and overflow incontinence in the acute phase of spinal cord injury is paramount. It involves preventing bladder over-distention and ensuring bladder emptying but also managing the dog as a whole and implementing hygiene measures that will prevent complications. The spinal patient might be especially susceptible to developing complications such as decubital ulcers and urinary tract infection because of the combination of neurogenic incontinence (leading to increased moisture on skin), recumbency [leading to reduced tissue perfusion and pressure >60 mmHg in risk zones such as scapula-humeral articulation, the greater trochanter and the thirteenth rib (44)] and reduced immunity after spinal cord injury [which is known to occur in people from decentralization of the autonomic nervous system (45)] potentially sustained by poor nutrition. Although these factors have not been clearly studied and established as “risks” in dogs after spinal cord injury, they are in people and for the time being, it appears logical to consider these as such. Guidelines for the management of urinary retention and incontinence in the acute phase are proposed in Table 1.

Table 1Acute phase of spinal cord injury—in hospital management (within 1 month of injury).

**Table:** 

**Checks/parameters**	**Bladder size**	**Bladder emptying**	**Feces emission**
Frequency	Every 4 h; and check fur is dry	Every 8 h or pending bladder size check	Suprasacral lesions: no need for rectal emptying but clean fur and skin if needed with checks every 8 h
			Sacral lesions: likely weak sphincters and constant fecal incontinence requiring frequent checks (e.g., every 4 h) and bath fur and skin then dry
Method	Ultrasound > manual palpation	Manual bladder expression > indwelling Foley catheter > intermittent sterile catheterisation; culture urine at removal of indwelling catheter or if suspicion of urinary tract infection: active urine sediment on urinalysis defined as > 5 white blood cell/high power field ± bacteriuria (>105 CFU/mL), pyuria, urine cloudiness and foul smell, pyrexia [see (46) for further information]	Inspection of animal
Cut-off/recommendations	Bladder volume estimation from ultrasound = L × W × ([DL + DT]/2) × 0.625 where L is longitudinal bladder length, W is transverse bladder width, DL is longitudinal bladder depth, and DT is transverse bladder depth [see (22)]; emptying proposed if estimated volume > 10 mL/kg	Consider factors that might impair manual bladder expression (e.g., non-cooperative patient, other soft tissue injuries, pain, untrained staff) or factors that might predispose to pressure sores if there is urine leakage (e.g., dogs with long fur)—in these instances consider indwelling Foley catheter	Dedicated neurology ward with shower station

**Table:** 

**Checks/parameters**	**Feeding plan**	**Skin and bed check**	**Change of recumbency**	**Blood pressure**
Frequency	Check body weight daily; offer food early on with meals every 8 h of a highly digestible (gastrointestinal) diet	Every 6 h	Every 6 h	Twice daily in cases with high thoracic lesions; autonomic dysreflexia reported in humans and experimental rats
Method	By mouth; offer food and water by bringing bowls to the animal's head because recumbent animals might not reach bowls in larger cages; avoid lateral recumbency after a meal	Inspection of animal in particular pressure points/bony prominences at risk such as scapula-humeral articulation, the greater trochanter and the thirteenth rib [see (44)]	Trained staff; ideally two members of staff	Cuff or Doppler measures
Cut-off/recommendations	Monitor urine analysis and biochemistry values in case of unexplained anorexia; search possible concomitant diseases (e.g., hypothyroidism, hypertension, protein losing nephropathy)	Provide absorbent bed pads such as those used for puppy toilet training; provide pressure-relieving mat; static pressure relieving mats generally insufficient; safe pressure in risk zones <60 mmHg [see (44)]	Provide whole body harness with handle, and washable sling support; remove when dogs in cage; See harness details in Table 3	Investigate if > 160 mmHg

### Mechanical Techniques to Manage Bladder Emptying

There are three proposed methods employed to empty the bladder in dogs following spinal cord injury: (i) manual emptying; (ii) intermittent aseptic catheterization; and (iii) placement of a indwelling catheter.

Manual emptying is the preferred method because it is quick, simple, cost effective and non-invasive. However, it is not always feasible if the patient is in pain, not cooperative or it might appear a challenging task for untrained staff. Care should be taken in cases with concomitant trauma to the abdomen and therefore potentially trauma to the bladder. A distended bladder might also be painful, rendering manual expression even more difficult. Although manual expression usually allows to empty “some” urine from the bladder, it's success can be extremely variable. It has been shown that on average only ~50% of the urine is removed by manual emptying, therefore leaving a large residual volume for some dogs, while it is efficient for others (47). Assessing residual volume with ultrasound might therefore be useful, especially in animals that are recovering to see how well they are voiding and try to determine if the urine in the cage is overflow or voiding. The impact of leaving a large residual volume in the bladder is not known. The presence of hematuria in 50% of cases managed this way suggests trauma to the bladder wall but whether this is due to the manual palpation or an effect urine stasis/large residual volume is not known (48). However, it remains likely that this technique is sufficient to prevent bladder over-distension, at least.

Intermittent sterile catheterization is also simple, guarantees an almost complete bladder emptying. But it is more invasive, carries a risk of introducing an infection in the bladder, can potentially cause urethral inflammation and stricture [as known in humans (49)] if done frequently, and might also appear challenging to untrained staff in particular in female dogs. In female dogs, the clinicians' preference might also often be to leave indwelling catheter in place rather than repeating sterile catheterization.

Placement of an indwelling catheter has similar advantages and disadvantages with the previous method but can lead to bladder mucosa minor trauma and bleeding (silicone Foley catheters are preferred), therefore blocking the catheter. It is usually used for a short time during hospitalization because it will be difficult for the owner to manage it at home. The use of a closed bag system and long-term placement of the catheter removes the need for repeated catheterization and is comfortable for the nursing team and the dog, also reducing the risk of urine scald especially for large dogs.

The prevalence of urinary tract infection in the acute phase of thoraco-lumbar spinal cord injury was first assessed in 2006 by Stiffler et al. in 92 cases (managed by manual emptying or catheterization) and found 15% of dogs with a urinary tract infection before surgery and between 12 and 20% of dogs with a urinary tract infection in the 7 days post-operatively, despite antibiotic prophylaxis (50). Non-ambulatory females and dogs with intra-operative hypothermia (<35°C) were at higher risk of developing a urinary tract infection. A year later, Bubenik et al. (51) described that 40% (44 of 105 dogs) of dogs catheterized after spinal cord injury had a urinary tract infection and the odds of developing one increased after each day spent in hospital. In that study, dogs had an indwelling urinary catheter managed with a closed catheter system (as opposed to intermittent clean/sterile) and had to have had a urine culture submitted when the catheter was removed, suggesting a possible bias toward those dogs with a clinically suspected infection but not urine culture performed. The concomitant use of antibiotics with an indwelling catheter was also found to increase the risk of urinary tract infection by 454% (OR, 4.54; 95% CI, 1.83–11.27) (51), an important finding signaling that the systematic use of antibiotics in this situation should be strictly avoided. Administration of dexamethasone within 48 h before surgery also constitutes an increased risk of developing a urinary tract infection in cases with acute spinal cord injury (52). Bubenik and Hosgood also looked at the risk of developing a urinary tract infection with each of the three methods described above to empty the bladder (53). There was no significance difference between the three methods (16% with manual emptying, 32% with indwelling catheter and 8% with intermittent catheterization), but the duration of bladder management dictated the risk of developing a urinary tract infection. In this study, there were many more infections in dogs with indwelling catheters than control dogs, which likely reflected the longer duration of therapy in dogs with indwelling catheters than dogs managed with manual expression or intermittent catheterization. This risk could be managed by earlier removal of the indwelling catheter. Olby et al. (48) followed dogs for 3 months after injury and found that 10 of 25 dogs developed a urinary tract infection during this time period, with most occurring between weeks 1 and 6. Of these cases, many had occult infections, now termed “bacteriuria”, of questionable clinical significance. During this study, owners were asked to monitor their dog's urine with dipsticks with the hope of identifying an easy method to alert owners to a developing urinary tract infection. This attempt was a failure, with most owners finding it extremely challenging to monitor urine in this way. The same group also found no effect of cranberry extract given in the acute phase of spinal cord injury on the occurrence of bacteriuria in a randomized control trial in dogs (54).

The reader is referred to previous reviews for a more in depth discussion around detection and treatment of urinary tract infections (55) and some guidelines are also presented by Baigi et al. (46). Briefly, a urinary tract infection is suspected if there is an active urine sediment, defined as > 5 white blood cell/high power field ± bacteriuria (>10^5^ CFU/mL), pyuria, urine cloudiness and foul smell and/or pyrexia.

### Pharmacological Interventions for Bladder Management

Pharmacological interventions will vary depending on the localization of the inciting injury and it is imperative to consider carefully the characteristics of the clinical signs when choosing medications. Table 2 present some possible medications for neurogenic bladder management.

**Table 2:** Possible drugs suggested to act on the lower urinary tract physiology during neurogenic dysfunction.

**Drug**	**Class/action**	**Dose**	**Common side effects**
Bethanechol	Parasympathomimetic/increases detrusor contractility	1.25–25 mg/kg p.o. q8h in dogs; 0.625–5 mg/cat p.o. q8h in cats; (titrate dose upwards to avoid side effects)	Vomiting, diarrhea, salivation, bradycardia
Oxybutynin	Muscarinic receptor antagonists/decrease detrusor contractility	0.5 mg p.o. q8-12h	Anticholinergic signs: reduced gastro-intestinal motility, dry mouth, tachycardia
Mirabegron	β3 adreno-receptor agonists/decrease detrusor contractility	<0.3 mg/kg p.o. q24h (side effects seen in dogs with a single dose of 0.3 mg/kg p.o.)	Tachycardia, arrythmias, erythema, vomiting, destruction of the zygomatic salivary gland
Phenylpropanolamine	Sympathomimetic/increase urethral tone	1.5 mg/kg p.o. q8-12h in dogs and cats	Hypertension, urine retention
Prazosin	Alpha 1-sympatholytic/decrease urethral resistance	1 mg/dog p.o. q8-12h under 15 kg and 2 mg/dog p.o. q8-12h above 15 kg in dogs; 0.25–1 mg/cat p.o. q8-12h in cats	Hypotension (syncope), salivation, sedation
Phenoxybenzamine	Non-specific alpha-sympatholytic/decrease urethral resistance	0.25–1 mg/kg p.o. q8-24h for minimum 5 days in dogs; 0.5–1 mg/kg p.o. q12h for minimum 5 days in cats	Hypotension (syncope, weakness)
Diazepam	Benzodiazepine/decrease urethral resistance	0.25–1 mg/kg p.o. q8-12h in dogs; Avoid in cats	Sedation, hepatocellular necrosis in cats
Dantrolene	Calcium release inhibitor in muscle/decrease urethral resistance	1–5 mg/kg p.o. q8h in dogs; 0.5–2 mg/kg p.o. q8h in cats	Rare—weakness, hepatoxicity, vomiting, hypotension

With a lesion of supra-sacral segments and in the acute phase of spinal cord injury, the clinician often faces a bladder that does not contract and is difficult to express due to persistent or increased sphincter tone. In that scenario, it would be ideal to be able to stimulate detrusor contractions with parasympathomimetic drugs such as bethanechol of carbachol, although this would also reduce the ability of the bladder to store urine. Bethanechol should only be administered in combination with medication that reduce sphincter tone such as alpha-sympatholytic drugs. However, bethanechol does not seem to change either urethral or bladder pressure in normal dogs (56) and is reported in a textbook as ineffective in a fully are flexic bladder (5) and therefore does not present a clear benefit for supra-sacral lesions. Parasympathomimetic molecules can also potentially cause increased motility of the gastrointestinal tract and diarrhea, which can render patient management difficult. Therefore, bethanechol should be kept as a second option if alpha-sympatholytic drugs have failed to help first. Indeed, alpha-sympatholytic drugs can be used in supra-sacral lesions to reduce sphincter tone which is typically increased or at least persistent and impairs voiding in particular when manual bladder expression is attempted. Alpha-sympatholytic drugs (e.g., prazosin, alfuzosin, and phenoxybenzamine) can be used in that respect and effectively target and relax the smooth urethral sphincter in normal dogs (57) but can cause hypotension. Further, striated muscle relaxants (e.g., diazepam, dantrolene, and baclofen) can be used to reduce the external urethral sphincter and assist urine voiding. Although combining a sympatholytic molecule and a muscle relaxant is attractive (and this might facilitate manual bladder expression), the utility of combining prazosin or diazepam in the urinary incontinence management of dogs with T3-L3 intervertebral disc herniation has recently been tested and the authors found no effect of these molecules on the urinary incontinence or duration of hospitalization (58). This was a retrospective study on 71 dogs with various injury grades and therefore of low power. It also seems clinically that drugs to facilitate manual evacuation (e.g., prazosin, diazepam) are often no longer needed after a few weeks.

Terazosin, a long-acting selective α-1 adrenoreceptor blocking molecule has been used to treat vesico-sphincter dyssynergia in spinal cord-injured male humans and reduced bladder outlet obstruction (59) but causes side effects such as collapse and has not been reported in clinical papers since 2002. In dogs, terazosin has been used to treat vesico-urethral reflex dyssynergia but not in the context of spinal cord injury and showed side effects in 93% of the cases (60). Similarly, tamsulosin is also a α-1 adrenoreceptor blocking molecule with higher affinity that terazosin (61) but has not been trialed after spinal cord injury in dogs.

When faced with an atonic bladder (either through a L7-S3 lesion causing a lower motor neuron bladder or because the bladder has been overstretched), the clinician has few options and there is no clear pharmacological method to reduce constant urinary leakage, which is problematic. Bethanechol can be trialed to improve bladder contraction but there is no clinical evidence to back this and the efficacy is poor in the author's experience.

## Management of Urinary Incontinence in the Chronic Phase

Guidelines for the management of urinary incontinence in the chronic phase are proposed in Table 3.

Table 3Chronic phase of spinal cord injury—in hospital management (weeks to months after injury).

**Table:** 

**Checks/parameters**	**Bladder emptying**	**Feces emission**
Frequency	Every 6 h; take dog first thing in the morning	Suprasacral lesions: no need for rectal emptying but clean fur and skin if needed with checks every 8 h
		Sacral lesions: likely weak sphincters and constant fecal incontinence requiring frequent checks (e.g., every 4 h) and bath fur and skin then dry
Method	Manual bladder expression > intermittent sterile catheterisation; possible implantation of a sacral anterior root stimulator for cases with T3-L3 spinal cord lesions and an upper motor neuron bladder	Feces emission usually spontaneous; light perineal stimulation (either digital wearing a disposable glove, or with a Q-tip) possible to trigger defecation; the sacral anterior root stimulator available for dogs with T3-L3 lesions will allow rectum emptying
Recommendations	Avoid indwelling Foley catheterisation as home method of management; Owners should monitor for the following signs and contact their veterinarian for a urine culture if any are seen: 1. A change in urination frequency, or a change in level of continence. 2. Pain on manual expression or during urination. 3. Foul odor to urine. 4. An increasing in licking of penis/vulva. 5. An increase in drinking. Possible cystometry every 6 months to screen for bladder overactivity (leading possibly to vesico-ureteral reflux) or bladder atony.	Regular drying; avoid wet fur; do not use talk powder; in female, inspect vulva carefully twice daily; diapers overnight can keep contamination down but prefer avoiding their use if possible

**Table:** 

**Checks/parameters**	**Feeding plan**	**Skin and bed check**	**Change of recumbency, mobility**
**Frequency**	Check body weight weekly in the first 3 months after spinal cord injury	Every 6 h	Every 4–6 h
Method	By mouth; offer food and water by bringing bowls to the animal's head because recumbent animals might not reach bowls in house if not able to move freely; avoid lateral recumbency after a meal	Inspection of animal in particular pressure points/bony prominences at risk such as scapula-humeral articulation, the greater trochanter and the thirteenth rib [see (44)]	Walking, physiotherapy, rehabilitation plan to be adapted to each animal pending the type of spinal cord injury and surgery the animal has received (presence/absence of implant, extent of spinal cord decompression, lesion level)
Recommendations	Low residue diet that reduces stool volume and creates firm stools; acid-suppression with for example proton-pump inhibitors if regurgitations, reflux are observed; possible use of probiotics; water intake should be of minimum 50 mL/kg	Provide absorbent bed pads such as those used for puppy toilet training; provide pressure-relieving mat; static pressure relieving mats generally insufficient; safe pressure in risk zones <60 mmHg [see (44)]	Provide whole body harness with handle (the top part of the harness should rest on the whither, the ventral part on the sternum, with the connecting front straps wrapping around the distal cervical region, avoiding contact with the shoulder joint, and the caudal straps resting caudally to the triceps muscle without interfering with the armpit or rubbing on the caudal shoulder muscles during the caudal phase of the stride); Provide washable sling support

### Mechanical Interventions

These are very much the same as in the acute phase although the different methods of bladder emptying have not been compared and their impact on occurrence of urinary tract infection has not been studied. In our experience, it seems that most owners will eventually manage bladder emptying by manual expression well and very few opt for repeated sterile catheterisation. We know from a study of 26 dogs (3) that the management of the incontinence (urinary and fecal) in the chronic phase (>3 months) of spinal cord injury requires owners to spend a median of ~3 h per week (ranging from 0 to 16 h) whereas the time required to manage mobility issues is a median of 10 h (ranging from 1 to 30 h), perhaps reflecting that incontinence is not a major problem for the owner, who tends to focus on locomotion. Further, in this study, 77% of owners felt that the effort in caring for their pet was worthwhile, although this is a population of owners who had decided to keep their dog alive in the chronic phase of spinal cord injury. Bauer et al. made similar observations in 30 chronically paralyzed and incontinent dogs where 60% of owners reported suspected urinary tract infections (although this could have been simply hematuria) but felt that this was not a problem and 35% reported them as “very infrequent” (62).

More recently, a high rate of bacteriuria was identified in dogs with chronic (>3 months) spinal cord injury (46). The most common isolate was *E. Coli*: 35 of 47 dogs had at least 1 positive urine culture and 13 dogs had recurrent bacteriuria. Fever and cloudy urine were not associated with infection, whereas pyuria was. Interestingly, of 35 dogs for which long term survival data was available, eight had died and only one was euthanized because of inability to empty the bladder. But no death seemed directly related to urinary tract dysfunction such as pyelonephritis or septicemia. This is in contrast with people with chronic spinal cord injury in which the rate of urinary tract infection is also high, ranging from 10 to 68%, but this is associated with a death rate of ~9%, perhaps reflecting the long duration of survival (decades) after the injury compared to a few years with dogs (63, 64). Although the data remain scarce in dogs and from a population of dogs referred to specialist hospitals, the above observations raise interesting questions. In particular, is it possible that dogs are more resistant to developing clinically important urinary tract complications than humans? It also raises the issue of the difficulty of determining a clinically relevant infection in this population of dogs. In humans, the most common signs of impending urinary tract infection are fever, increased spasticity and pain, signs that the patient can report. To help detecting the occurrence of urinary tract infection, a questionnaire has been developed by Prof. Olby in an attempt to gather the information needed to discern clinically important infection that should be treated (Appendix 1) from those that would not require medical attention. This questionnaire was designed for the owner of the dog.

Finally, it would also be interesting to test the effect of urine acidifiers (ammonium chloride) or antiseptics (in particular nitrofurantoin or methanamine) to prevent the development of urinary tract infection in the chronic phase of spinal cord injury in paraplegic dogs.

### Pharmacological Interventions for Bladder Management

In the chronic phase of supra-sacral spinal cord injury, the bladder tone might increase independently from brain control, leading to involuntary detrusor contractions, although this type of dysfunction does not seem to be a common referral request or complaint from owners. Perhaps it facilitates bladder emptying in some cases. In that instance, muscarinic receptor antagonists (e.g., tarafenacin, oxybutynin, imidafenacin, propioverine) can be used. In particular, the bioavailability of imidafenacin in dogs is known (65) making it a potential drug candidate to test. But there is no report of their use in pet dogs and these molecules can come with marked adverse effects in humans (dry mouth, bradycardia followed by tachycardia, arrythmias, constipation, blurred vision etc.). Therefore, more targeted detrusor relaxants causing fewer side effects have been researched. In particular, β3 adreno-receptor agonists have emerged as cleaner molecules in humans and are known to be safe in dogs, such as mirabegron (66); solabegron can relax the bladder while increasing the micturition reflex in experimental dogs (67). These drugs might benefit pet dogs in the chronic phase of spinal cord injury but have not been used in the clinic so far.

Local delivery of drugs constitutes another option. Botulinum-A toxin injection into the detrusor muscle via cystoscopy was first described in 2000 in humans (68) and is an effective way to inhibit acetylcholine release from parasympathetic nerves resulting in a decrease in bladder overactivity and increase in bladder capacity, altogether greatly reducing leaking episodes (69). This technique applies best to overactive “upper motor neuron” bladders and is in theory feasible in the clinic in dogs with chronic paraplegia and frequent emission of spurts of urine due to involuntary detrusor contractions. It has only been tested once in female dogs with non-neurogenic incontinence with good results (70).

### Surgical Interventions

Surgical interventions to help improve the clinical signs and management of urinary incontinence can be separated in those that focus on the effector organs, the bladder and/or sphincters, and those that exploit viable peripheral nervous structures below the lesion.

In the sub-acute to chronic phase of spinal cord injury, placement of drainage systems such as low-profile cystotomy tubes might allow comfortable drainage of the urine for the dog and the owner in the few weeks after injury while waiting for potential recovery. Although this requires surgery and can lead to bladder infection, it remains a simple and relatively non-invasive procedure that can ensure complete bladder emptying and seems well rated by owners (71). Aside from this option, there are no reports of surgeries on the bladder itself in companion dogs with spinal cord injury, whereas bladder augmentation is commonly used in people (72) to improve bladder capacity. This might reflect a low demand from owners of affected dogs for advanced treatment of the incontinence, often content with their management or reluctant to put their dog through more surgical interventions. Our lack of experience in that field means that we are currently unable to describe to owners the potential risks or benefits of bladder surgeries aimed at improving incontinence. Some of these, such as vesicostomy are certainly feasible as described in experimental dogs (42). Vesicostomy limits the development of detrusor hypertrophy, which could lead in the long term to total lack of urine storage but seems to come with a number of issues such as bladder infection, surgical failure or skin irritation.

For sacral lesions, the challenge to restore continence is greater than with supra-sacral injuries because of the loss of neurons or peripheral nerves leading to weak sphincters and constant leakage. To circumvent this, two avenues have been researched: re-innervating or re-constructing the urethral sphincter. Re-innervation can be done with peripheral nerve transfer: femoral to pudendal nerve transfer (73), obturator to pelvic and sciatic to pudendal nerve transfers (74–77) have been done in experimental dogs with promising results but have not reached the clinic. These procedures seem to have merit and are probably worth exploring in the future. Re-construction of the bladder neck and urethra in dogs appears in a textbook (5) suggesting that this can be successful but no data are published. There might also be a place for implantation of hydraulic occluders (i.e., artificial urethral sphincter) that are able to restore urethral pressure (78). A good wealth of experience has been gained with this method over recent years for the treatment of urethral sphincter mechanism incompetence (79) which could potentially be exploited in the field of canine spinal cord injury.

### Functional Electrical Stimulation

Dogs have been employed for a long time in neuroscience to study the pathophysiology of the bladder after spinal cord injury and ways to restore micturition. From the seventies mainly, spinal cord transection models (80) seemed to re-create in dogs the state of bladder denervation observed in humans (we are providing some references here in footnotes because these are not easily accessible to us but might be to other scientists—see Footnote^1^), although the data was published in Russian, Japanese or French and is not readily accessible. These transection models, possibly ethically debatable, are still often used in China and Japan and a vast amount of data are available (81).

Various approaches have been used to improve bladder function and restore normal continence, and functional electrical stimulation has been one of the major lines of research followed (81, 82). Experiments in dogs have included stimulation at different levels including transcutaneous bladder stimulation (83), pelvic nerve stimulation (84) or pudendal nerve stimulation (43, 85) outside the spinal canal or intraspinal sacral roots stimulation (25, 86).

These techniques are often quite convincing [e.g., pudendal nerve stimulation delays bladder fibrosis and improves continence in dogs after spinal cord injury (43)] but have rarely converted into clinical applications, perhaps due to a perceived invasiveness, cost or possibly because the reported data do not reach the right audience. On the contrary, it seems that the data gathered in dogs has successfully been implemented in the clinic to humans with spinal cord injury (81) stressing the need to revisit the applicability of these studies to companion dogs.

**Figure 1:**
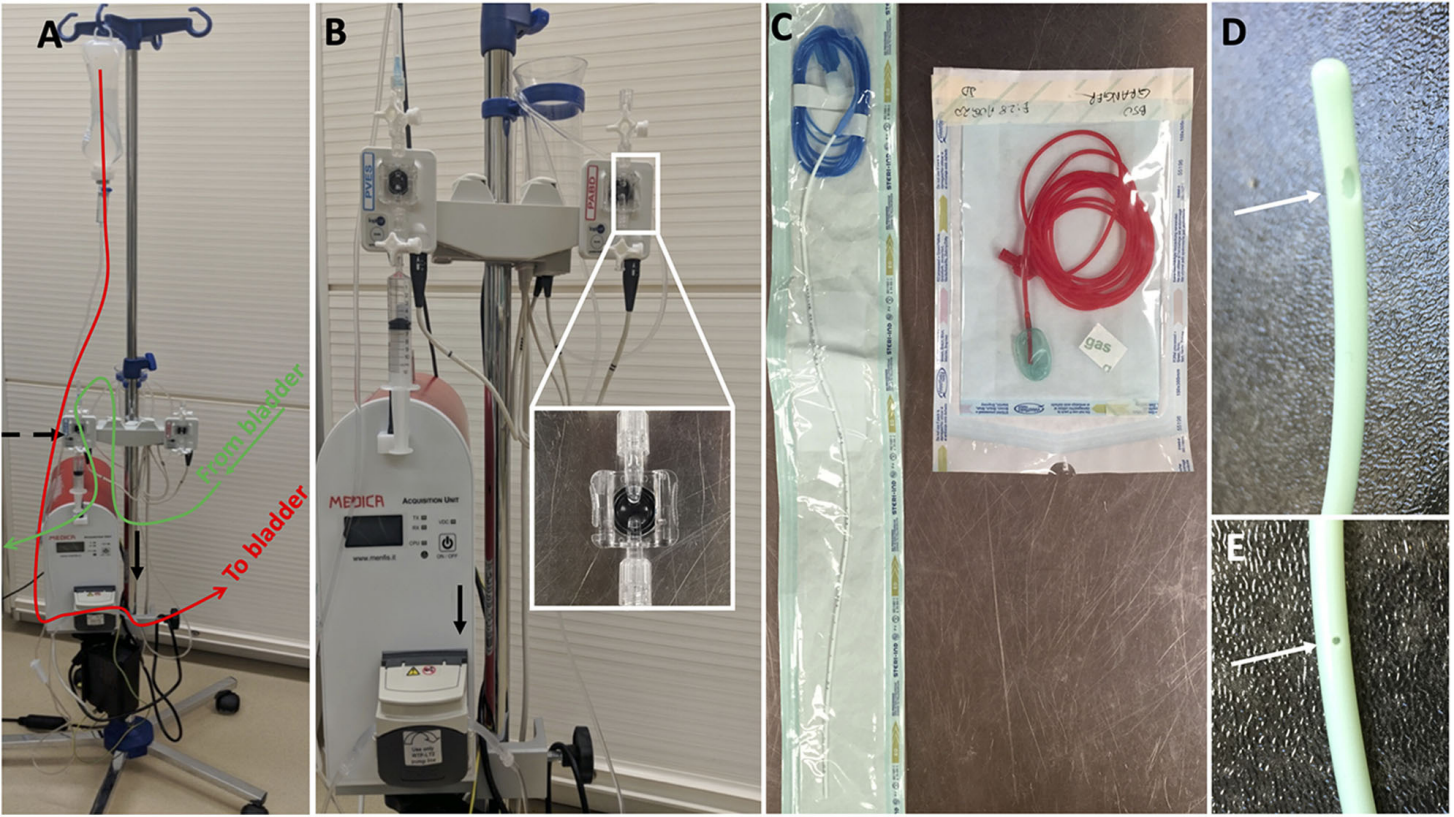
Urodynamic equipment to perform cystometry in dogs; **(A)** it is composed of a pump (black arrow) infusing sterile fluid into the bladder through a dual lumen catheter placed in the bladder (follow red line); the dual lumen catheter measures water pressure in the bladder which is recorded at the level of a pressure transducer (black dashed arrow) connected to a computer software; **(B)** shows the pump more closely and the two pressure transducers typically used to measure bladder pressure and rectal pressure; **(C)** on the left a dual lumen catheter with one lumen used to infused sterile saline in the bladder (transparent port) and one lumen used to measure pressure in the bladder (blue port with blue line extension); on the right a rectal catheter used to measure indirectly abdominal pressure (see **Figure 3** for related pressure curves); **(D,E)** show the two ports of the dual lumen catheter, one large at the tip allowing sterile saline infusion and one 5 cm caudal, smaller and measuring fluid pressure.

One striking example of the knowledge gained from canine experiments is sacral nerve stimulation, described in the 19,000 and later in several studies (83, 87, 88). This technique was very successful and translated from bench to bed in <15 years following work from Brindley who developed a human sacral anterior root stimulator (89). This system consists of placing implants along the pairs of sacral nerves via a lumbo-sacral laminectomy. The implants are connected to a sub-cutaneous receiver that the patient can activate with a transducer. The transducer needs to be positioned above the skin under which the receiver is located, and a remote control delivers the electrical current to the transducer to activate the system. This provides nearly complete bladder emptying, solving the problem of lack of voiding. It also restores continence when the sacral sensory nerve roots are rhizotomised in surgery during implantation thereby preventing reflex incontinence (90). The Brindley's neuroprosthesis exploits the fact that the bladder is a smooth muscle, therefore relaxing slowly, and that the external urethral sphincter is striated, therefore relaxing quickly and functions by delivering short burst of stimulation intercalated by periods with no stimulation. Hence, during sacral nerve stimulation, both the smooth detrusor muscle and external urethral sphincter contract, leading to increase in bladder pressure but no emission of urine—this can be seen as a built-up pressure against a closed sphincter. Once the first short burst of stimulation finishes, the external urethral sphincter muscle relaxes quickly but the detrusor, being a smooth muscle relaxes more slowly, leaving a period of time during which the pressure in the bladder exceeds that of the sphincters leading to emission of urine. A series of on and off stimulations are then delivered until this “artificial dyssynergia” empties the bladder fully. The Brindley device has been translated to dogs successfully, a good example of “reverse” translation and described in 9 dogs in 2013 (91). The voiding efficiency of the system in dogs was >90%. Feces are frequently passed during bladder stimulation as well in implanted dogs, hence limiting loss of feces during the day, as is also observed in humans (92). The best candidates for this treatment are dogs with T3-L3 lesions and an upper motor neuron bladder that have not regained deep pain perception and locomotion. These animals can be implanted from a few weeks to years after their injury. Dogs receive the neuroprosthesis most commonly on the second pair of sacral nerves via a routine lumbo-sacral laminectomy (Figure 1). The first author of this review has now treated 25 dogs with this system. Finally, it does not appear needed in dogs to perform the sacral nerve sensory rhizotomy, although this is possible (93), and the efficiency of the system is such that if used regularly (e.g., four times in a day), the bladder remains small enough to prevent leakage of urine.

Finally, there is a view that stimulation of lower centers/motor neurons might re-train the bladder. Firing induced by electrical stimulation can be expected to alter circuitry strength and promote specific patterns of connectivity through Hebbian mechanisms. Indeed, recent experiments in rodents from the Edgerton laboratory confirmed that repeated electrical stimulation of the spinal cord, at frequencies of 40 Hz, changed neural networks controlling bladder function (94). Similarly, Grégoire Courtine and Daniel Eberli's laboratories found that multisystem neuroprosthesis developed to train locomotor function in rats also had an effect on bladder function (80). Data from humans and studies in rats showed that locomotor training exercises after spinal cord injury improved bladder function (95); however, the use of various orthoses as support systems rather than active forms of training neither improve the abilities to stand and walk nor improve the physiological health of humans with spinal cord injury, suggesting that active methods of exercise may have more physiological benefits for spinal cord injury subjects (96).

## Management of Fecal Incontinence

Guidelines for the management of fecal incontinence in the acute and chronic phases are proposed in Tables 1, 3, along with suggestions to manage the dog's environment and feeding.

The primary concern expressed by owners of dogs with fecal incontinence is management of the “mess” produced by inadvertent defecation, with secondary concerns of skin damage due to contamination with fecal material and contamination of the vulva causing urinary tract infections. Careful questioning of the owner is needed to determine when the incontinence is occurring and the nature and volume of the stool. The most practical and effective management technique is to use a low residue diet (e.g., gastro-intestinal/hypoallergenic diets) that reduces stool volume sometimes dramatically, and usually resolves loose stool or diarrhea. All dietary indiscretions should be avoided because the consequences can be challenging.

Once stool volume and consistency have been addressed, further management techniques can be used to reduce accidents. First, a bathroom routine should be established providing ample opportunities to defecate. Owners are advised to start by taking the dog out first thing in the morning for bladder expression. Typically, they will defecate at that time, but if not, some owners find light perineal stimulation (either digital wearing a disposable glove, or with a cotton-tipped applicator) can trigger defecation, as well as manual bladder emptying. In some dogs, a meal or exercise is necessary to trigger defecation and owners rapidly learn the best routine for their dog. The question of diapers becomes important for some. For example, in dogs that routinely defecate in their sleep, if they produce only small volumes of hard stool, the problem is easy to deal with in the morning. However, some owners find use of diapers overnight can reduce contamination of bedding. This is effective but skin health should also be considered, and an alternative is use of plastic backed “pee” pads (i.e., absorbent bed pads such as those used for puppy toilet training). Overall, the key is to reduce the volume and moisture of stool, resulting in a reduced frequency of defecation and producing stool that is easy to clean up.

Similar to dogs, management of gastrointestinal dysfunction in humans after spinal cord injury is often limited. While evidence for these interventions is lacking in dogs, they nevertheless can give some useful ideas for management of dogs with chronic spinal cord injury. Treatments for upper gastrointestinal dyspepsia include acid-suppression with proton-pump inhibitors and dysmotility along the entire gastrointestinal tract can be targeted with prokinetics. Pharmacological interventions targeting bladder overactivity with anticholinergics has become commonplace but carries the potential for unintended side effects of diminished gastrointestinal motility, as mentioned above. In addition to prokinetics, neurogenic bowel in humans is treated by increasingly invasive procedures. If diet and fluid management combined with manual evacuation are unsuccessful, individuals may resort to chemical stimulants containing glycerine or bisacodyl. Fluid management is problematic as individuals balance the need to bladder catheterize (lower fluid intake to reduce need for catheterisation) with demands of a bowel program (increase fluid intake to facilitate elimination). Extreme cases of colonic dysmotility may lead individuals to consider surgical procedures such as antegrade continence enema and colostomy, which are not reported in dogs in the context of spinal cord injury. Other people will benefit from sacral nerve stimulation via implanted devices (97). This is also possible in dogs with the sacral root stimulator described above (91): the system in dogs does allow stimulation of defecation (either using bladder voiding parameters or by setting parameters for bowel emptying), often leading to a routine of twice a day elimination of feces at the time the sacral stimulation is done for bladder emptying.

Another aspect that has been studied in humans and animals after spinal cord injury is a change in the gut microbiome which has been shown to be greatly altered after stroke (98) and traumatic brain injury (99). Only a few studies have investigated the spinal cord injury microbiome in both humans and rodent models and two treatment options have shown evidence of improving recovery after spinal cord injury (100, 101). Both probiotics and melatonin have shown to restore some of the microbiota disturbances triggered by spinal cord injury; however, future research should focus on the temporal changes of the spinal cord injury-induced dysbiosis and consider that microbial shifts can also potentiate inflammation which is known to occur following spinal cord injury and possibly add to the chronic inflammatory state of spinal cord injury individuals.

## The Study of Urinary Incontinence With the Canine Translational Model of Spinal Cord Injury

**Figure 2:**
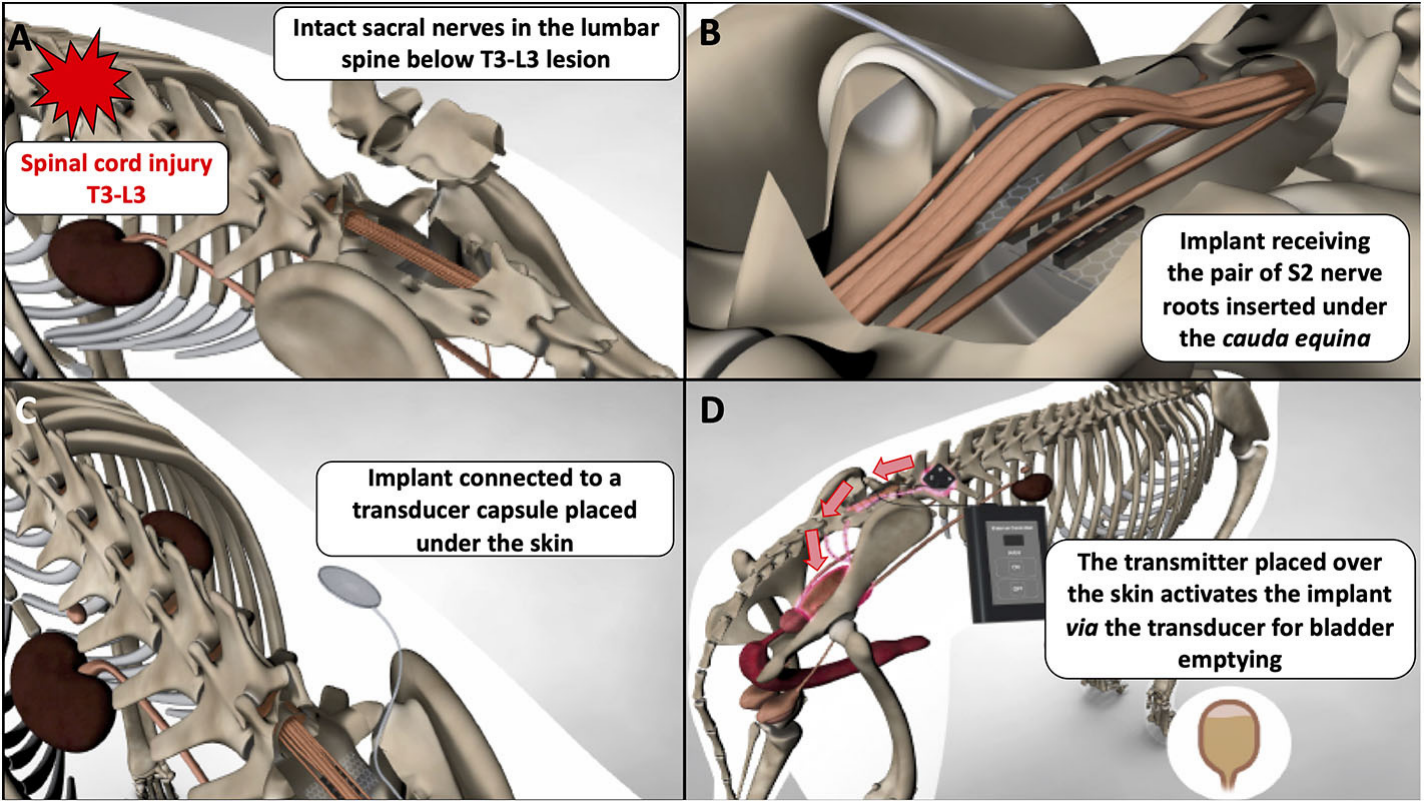
Schematic demonstrating placement and function of a canine sacral nerve stimulator for bladder emptying in chronically paraplegic dogs; **(A)** in dogs with T3-L3 spinal cord lesions, the sacral nerves below the lesion remain intact and can be accessed via lumbo-sacral laminectomy; **(B)** a “book” electrode containing two gutters can receive a pair of sacral nerves (e.g., the S2 pair) when the implant is slotted underneath the dural cone and *cauda equina*; **(C)** the implant is connected via a cable (named a Cooper cable) to a sub-cutaneous transducer that can be palpated by the clinician and the owner; **(D)** the transducer is activated with a remote system brought close to the skin and the transducer; this generates an electrical current that flows to the implant, stimulate the sacral nerves, and leads to efficient bladder emptying.

**Figure 3:**
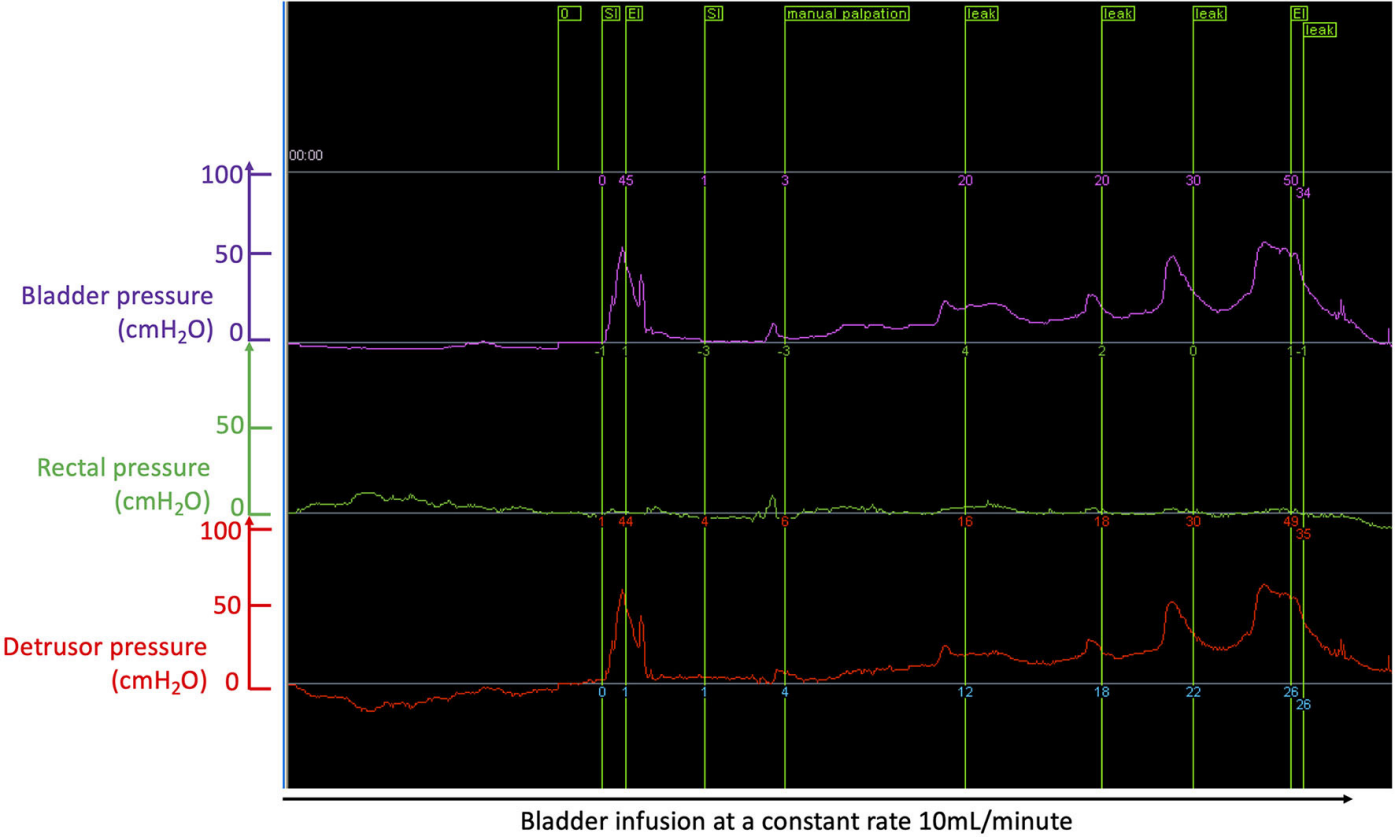
Cystometry curves recorded during bladder filling at a constant rate of 10 mL/min with a dual lumen catheter placed in the bladder through the urethra; the purple trace shows bladder pressure; the green trace shows rectal pressure measured from a rectal balloon; the red trace is the “true” bladder pressure or “detrusor” pressure obtaining by subtracting the bladder pressure (purple curve) by the rectal pressure (green trace): this allows correction for increase bladder pressure peaks due to increase in abdominal pressure, e.g., when the dog moves or barks. In this example of a dog with chronic severe T3-L3 spinal cord injury (causing paraplegia and incontinence), one can see a peak of pressure corresponding to manual palpation by the clinician (green flag at the top “manual palpation” used as a test control); the first peak of pressure to the left of the recording is an artifact; further to the right, involuntary peaks of pressure are recorded and flagged (see green flags at the top “leak”) and lead to involuntary emission of urine (i.e., incontinence); during filling, the detrusor pressure slowly rises (here to pressure >50 cmH_2_O); however, full voluntary emptying should occur in normal animals when the detrusor pressure reaches ~20 mH_2_O and this has not happened here.

Recovery of urinary continence and pelvic floor muscle function in the chronic phase of spinal cord injury is highly valued by humans (1, 2). Dogs have been proposed as a large animal model for this purpose in particular those with T3-L3 spinal cord injury resulting in “upper motor neuron” bladder dysfunction (102, 103). The physiology of urination in dogs is more likely to be closer to humans compared to rodents because they are “house trained,” and undergo larger fluctuations in bladder size pre-injury, having a similar number of micturitions per day to humans compared to rodents. Dogs are of larger size than rodents and the natural occurrence of spinal cord injury gives advantages to study bladder dysfunction over other animal models. It is possible to employ urodynamic techniques—such as cystometry or profilometry (Figure 2)—to study bladder physiology in dogs (104). This gives access to measures such as bladder compliance (i.e., the change of pressure for a given change of volume that describes the ability of the bladder to store urine), involuntary bladder contractions (Figure 3), pressure and volume at capacity, voiding efficiency etc. and these measures have been clearly defined by the International Continence Society (105, 106). As such, randomized control trials exploiting companion dogs to study the effect of promising interventions (druggable molecules or cell therapies) for chronic spinal cord injury repair have reported cystometry or continence outcomes. We are still at an early stage of describing the pathophysiological consequences of spinal cord injury on continence in dogs. Most studies of chronically paralyzed dogs did not document an improvement in continence (107–109), which may reflect the complexity of restoring long distance tracts responsible for brain controlled functions. An interesting study recently showed that sub-cutaneous injection of the broad-spectrum MMP inhibitor, GM6001, in the acute phase of the injury (<48 h) led to an increase in bladder compliance compared to controls at 6 weeks of follow-up (23). The pattern of voiding was however unchanged, and these dogs had incomplete spinal cord injury and recovered from their injury, constituting a paradigm different from the problem of reversing incontinence in the chronic phase. Nevertheless, it raises the possibility of investigating this medication in dogs with complete lesions in order to study the long-term effects and sustainability of the changes on urinary continence in the chronic phase of injury.

## Conclusion

Appropriate care of the lower urinary tract system in dogs after spinal cord injury is critical because this can affect quality and timing of recovery of function and the future life of the animal. Fortunately, most dogs with incomplete thoracolumbar spinal cord injury regain manageable continence, although mechanisms through which this occurs and methods by which this can be further improved upon remain unclear. Continued studies into the acute and also chronic changes of the lower urinary tract after spinal cord injury are important in order to better define the impact of incontinence on the animal and to understand changes over time that could be improved. Development of guidelines for treatment and careful recording of data on urinary tract function will benefit this population of dogs as well as provide much needed data for better understanding of this system after injury.

While evidence-based guidelines for the management of urinary incontinence in companion dogs are scarce, there is even less known about fecal incontinence in dogs. For example, pathological changes to the bladder or gut are largely unknown in companion dogs as are their consequences on function? Owners are frequently managing their dog's incontinence using “common sense” with little veterinary input and the incidence and importance of bacteriuria or urinary tract infections is unknown. While owners rapidly learn to cope with fecal dysmotility, it is very likely that our lack of understanding in the changes in autonomic function and microbiome result in a failure to recognize therapeutic opportunities that could improve the quality of life of animals.

Finally, it is possible that better characterization of autonomic dysfunction in companion dogs will reveal important knowledge that could be transferrable to humans with spinal cord injury. Here, companion dogs could play a pivotal role as a natural model of spinal cord injury and effectively complement other models of spinal cord injury.

## Author Contributions

NG, NO, and YN-L participated in manuscript conception, preparation, and editing with the first NG leading the conception and writing and creating the figures and tables. The additional members of the Canine Spinal Cord Injury Consortium (CANSORT-SCI) consortium contributed to manuscript conception, editing, and review. All authors contributed to the article and approved the submitted version.

## Conflict of Interest

NG is employed by the company CVS Ltd as a clinical neurologist and the Royal Veterinary College, University of London, as Senior Research Fellow. The remaining authors declare that the research was conducted in the absence of any commercial or financial relationships that could be construed as a potential conflict of interest.

## Footnotes

^1^Mokhort V. Findings on the restoration of bladder function in experimental spinal cord injury. *Vopr Neirokhir*. (1963) 27:38–41. Kondo A. The contraction of the ureter. II. Observations of the ureter of cord injured patients and cord transected dogs. *Nagoya J Med Sci*. (1970) 32:395–406. Vishnevskii A. Restoration of bladder function following injury to the spinal cord. (1971) 47:34–40. Sarramon JP, Lazorthes Y, Buffet J, Arbus L. Spinal cord stimulation and neurologic bladder. Experimental study and first clinical applications. *J Urol Nephrol (Paris)* (1973) 79:571–80.

## Appendix 1

**NC State Veterinary Hospital**

**Date: __________**

**Urinary Tract Health Information in Paralyzed Dogs**

**History:**

1. Has there been a recent change in the general mental attitude of your dog? □ Yes □ No. If yes, describe:__________________________________________
2. Has there been an increase in kicking, jerking, stiffness or spasms in the hind legs? □ Yes □ No. If yes circle the sign (s).
3. Has there been an increase in dribbling or inappropriate urination? □ Yes □ No
4. Is your dog licking its vulva or penis? □ Yes □ No
5. Is there a discharge from the vulva or penis? □ Yes □ No
6. Has the frequency of expressing/catheterization changed? □ Yes □ No If yes, provide details. ___________________________________________________
7. Has your dog been more painful recently? □ Yes □ No If yes, please explain and note location of pain:_____________________________
8. Have you noticed any signs of pain or discomfort while expressing your dog's bladder? □ Yes □ No. If yes, please explain: ___________________________________
9. Has there been a change in the smell of urine? □ Yes □ No If yes, please describe: ___________________________________
10. Has there been a change in the appearance of urine? □ Yes □ No. If yes, please describe: ___________________________________

**Physical exam:**

1. Body temperature
2. UTI specific physical examination findings
  □ Changed mentation □ Lethargy and malaise □ Painful flank and bladder region □ Increased spasticity
  Additional comments:
3. Urine appearance and odor: □ Cloudy □ Dark □ Bloody □ Foul odor
4. Discoloration of skin around vulva or penis □ Yes □ No
